# Supplementary material for: Honey bee food resources under threat from climate change
Source: Nat Commun. 2025 Dec 30;17:1331. doi: 10.1038/s41467-025-68085-6 (PMC12873215; doi:10.1038/s41467-025-68085-6)
Supplement: Supplementary file 1 — Supplementary Information [file 41467_2025_68085_MOESM1_ESM.pdf]

Supplementary Information for

Honey bee food resources under threat from climate change

Andreia Quaresma[1,2,3,4], Johannes M. Baveco[5], Robert Brodschneider[6], Willem Bastiaan Buddendorf [5], Norman L. Carreck[7], Kristina Gratzer[6], Fani Hatjina[8], Ole Kilpinen[9], Ivo Roessink[6], Flemming Vejsnaes[9], Jozef van der Steen[10], M. Alice Pinto[1*+], Alexander Keller[11*+]

[1]CIMO, LA SusTEC, Instituto Politécnico de Bragança, Campus de Santa Apolónia, 5300-253, Bragança, Portugal

[2]Departamento de Biologia, Faculdade de Ciências da Universidade do Porto, Rua do Campo Alegre, S/N, Edifício FC4, 4169-007, Porto, Portugal

[3]CIBIO, Centro de Investigação em Biodiversidade e Recursos Genéticos, InBIO Laboratório Associado, Campus de Vairão, Universidade do Porto, 4485-661 Vairão, Vila do Conde, Portugal

[4]BIOPOLIS Program in Genomics, Biodiversity and Land Planning, CIBIO, Campus de Vairão, 4485-661 Vairão, Vila do Conde, Portugal

[5]Wageningen Environmental Research, P.O. Box 47, 6700 AA Wageningen, the Netherlands

[6]Institute of Biology, University of Graz, Universitätsplatz 2, 8010 Graz, Austria

[7]Carreck Consultancy Ltd, Shipley, West Sussex, RH13 8GD, UK

[8]Department of Apiculture, Institute of Animal Science ELGO 'DIMITRA', 11145 Nea Moudania, Greece

[9]Danish Beekeepers Association (DBF), Fulbyvej 15, DK-4180 Sorø, Denmark

[10]Alveus AB Consultancy, Kerkstraat 96, NL-5061 EL Oisterwijk, Netherlands

[11]Cellular and Organismic Networks, Faculty of Biology, Ludwig-Maximilians-Universität München, Großhaderner Str. 2-4, 82152 Planegg-Martinsried, Germany

*Jointly supervising authors

[+]Corresponding authors: M. Alice Pinto (apinto@ipb.pt), Alexander Keller (keller@bio.lmu.de)

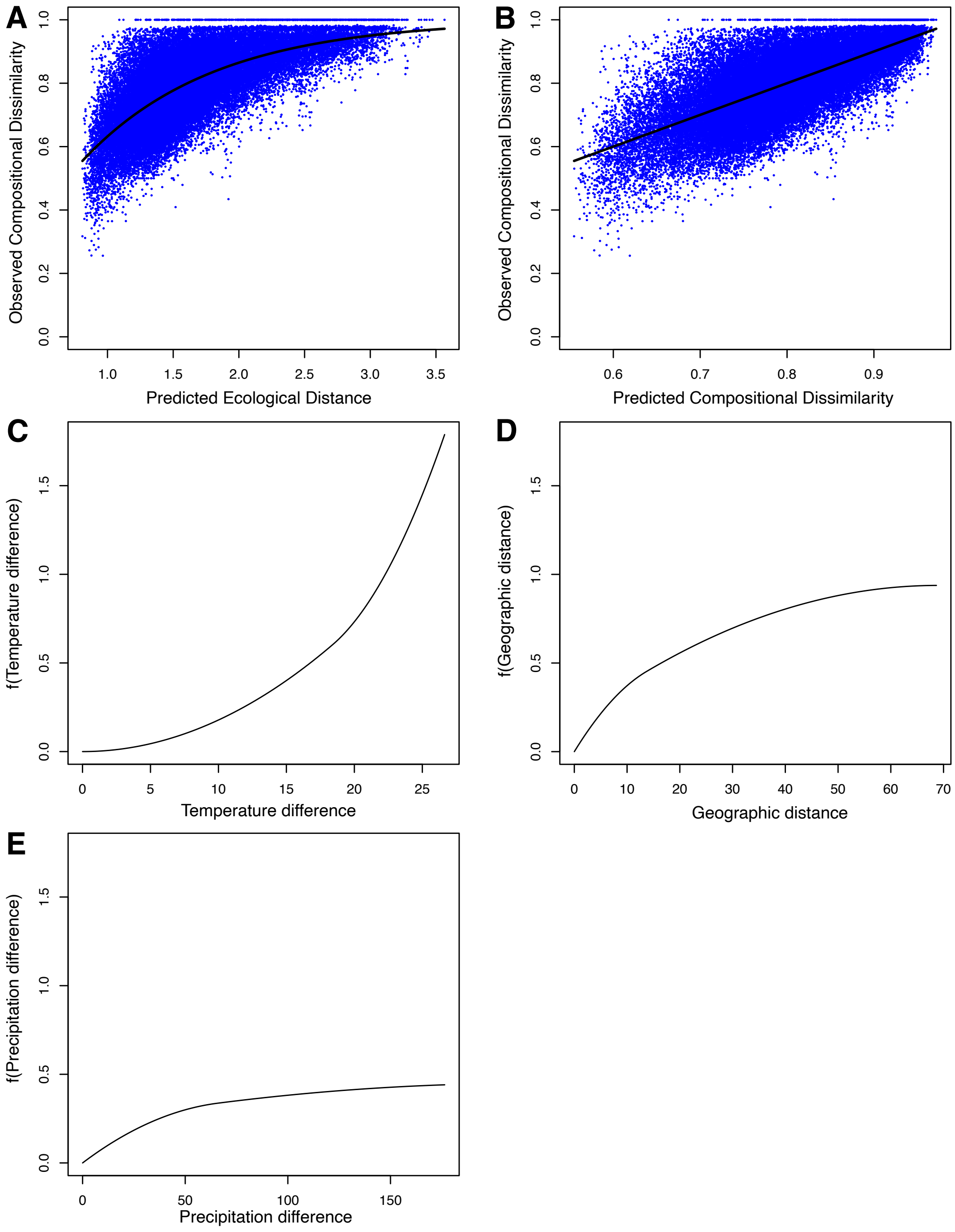

Supplementary Fig. 1. Generalised Dissimilarity Model (GDM) for each predictor variable tested (temperature, geographic distance, and precipitation). The model quantifies the relationship between compositional dissimilarity in pollen samples and environmental or spatial variables. Steeper slopes indicate a stronger effect of the predictor variable on compositional dissimilarity. The model explained 48.47% of total variance in pollen composition (pseudo-$F(3, 48\,205) = 0.52$, $p < 0.001$). **A** Observed compositional dissimilarity vs. predicted ecological distance, illustrating the overall model's fit in predicting dissimilarity based on ecological variables. **B** Observed compositional dissimilarity vs. predicted compositional dissimilarity, showing the comparison between actual dissimilarity values and model predictions. **C-E** reflect how specific environmental and spatial variables influence the variation in pollen composition across the studied locations, as **C** effect of temperature (Σ I-spline = 1.82) differences between locations on compositional dissimilarity, **D** the effect of spatial separation (geographic distance, Σ I-spline = 0.94) on dissimilarity in pollen composition and **E** the relationship between differences in precipitation (Σ I-spline = 0.44) and compositional dissimilarity in pollen samples.

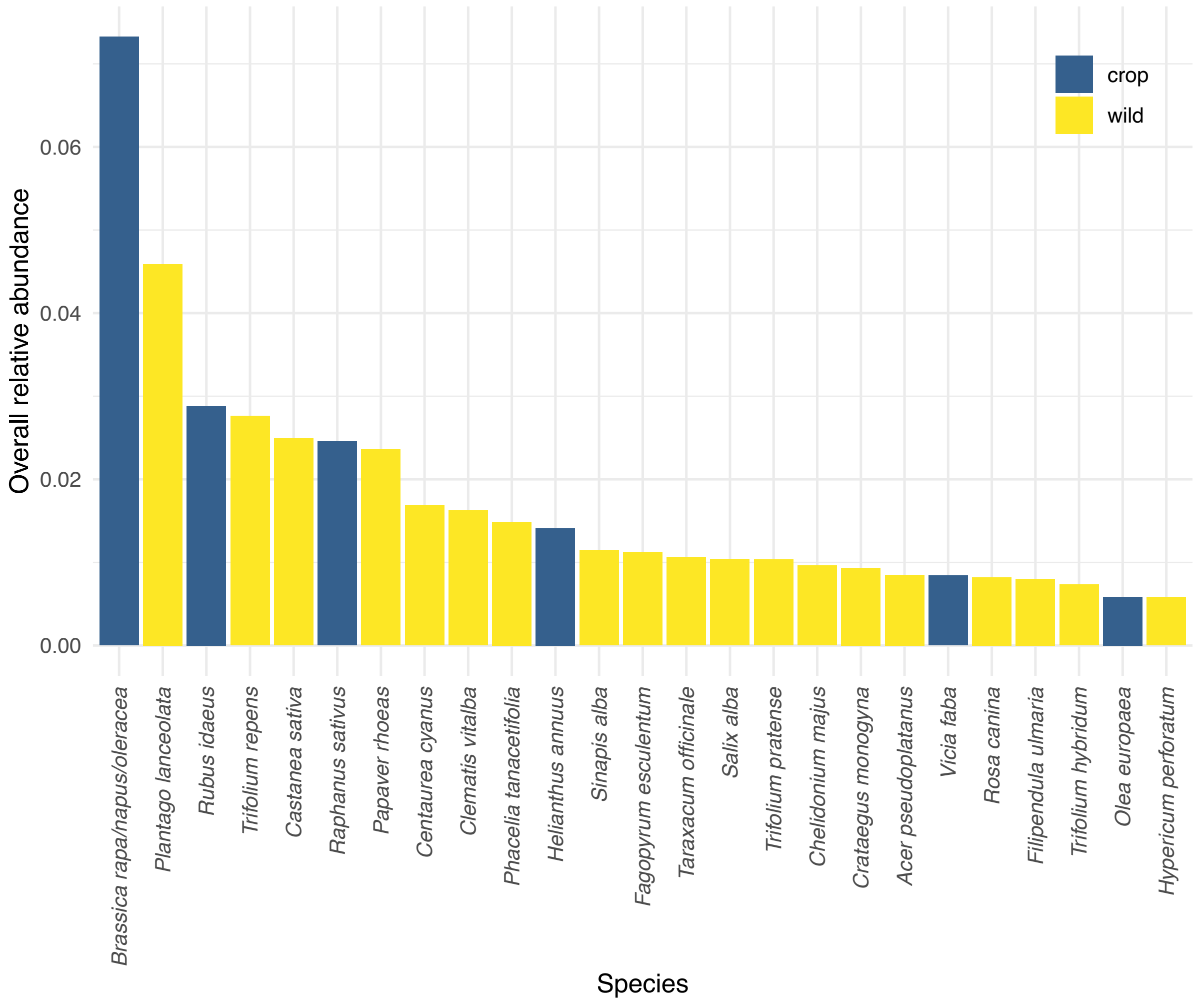

Supplementary Fig. 2. The most abundant 25 plant species in honey bee-collected pollen over the entire dataset. The RRAs are expressed over all sampling sites and time points accumulated.

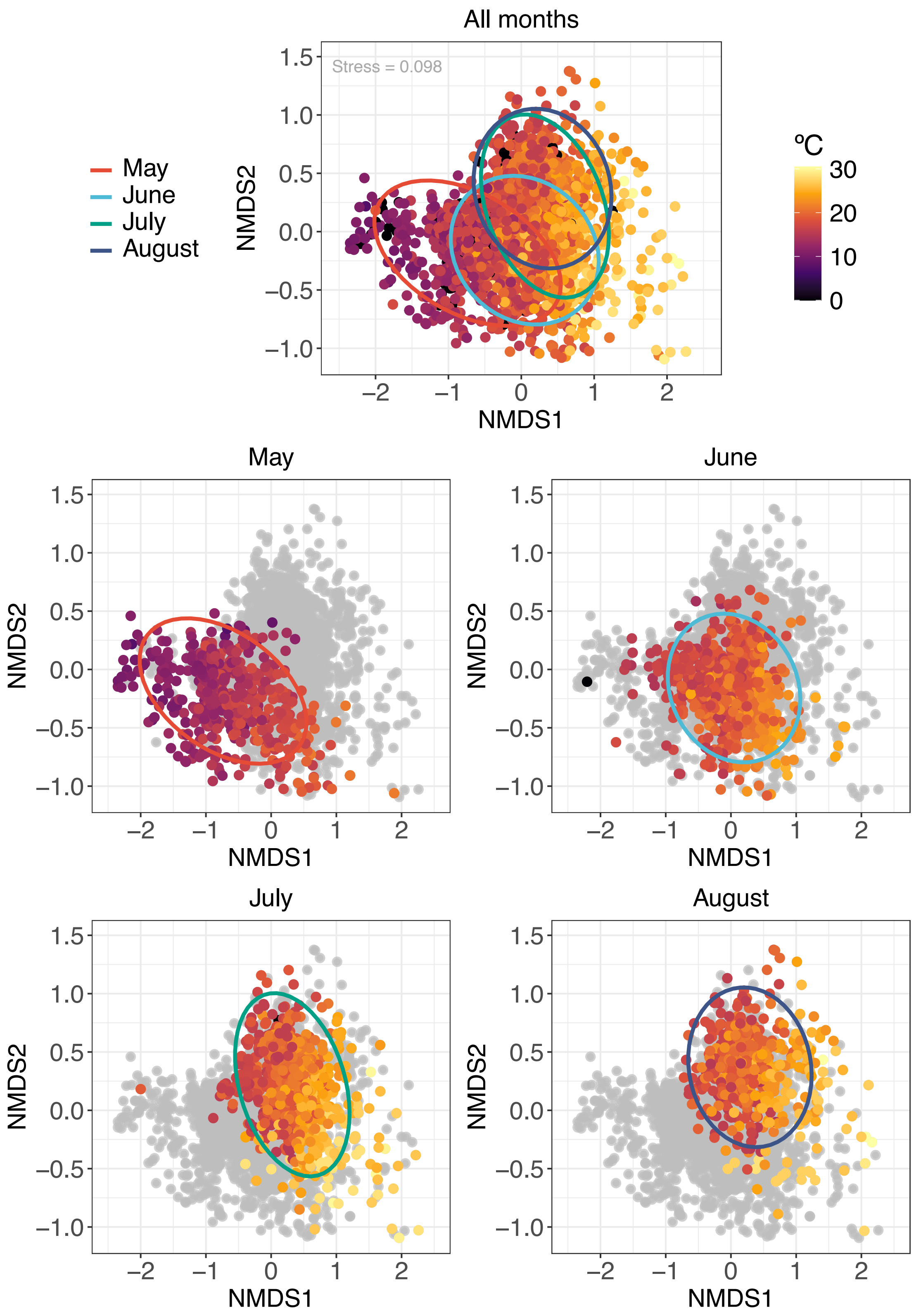

Supplementary Fig. 3. Non-metric multidimensional scaling (NMDS) of pollen composition coloured by temperature. The ordination is based on Bray-Curtis dissimilarity of n = 2 500 samples and includes five dimensions (k = 5), selected based on minimising stress. The final stress value was 0.098. Upper panel includes all data points, lower panels show a subset of samples from one month. The colour bar gradient represents the temperature in each sample, allowing visualisation of the difference between colder and warmer temperatures for the overall sampling sites and each sampling month.

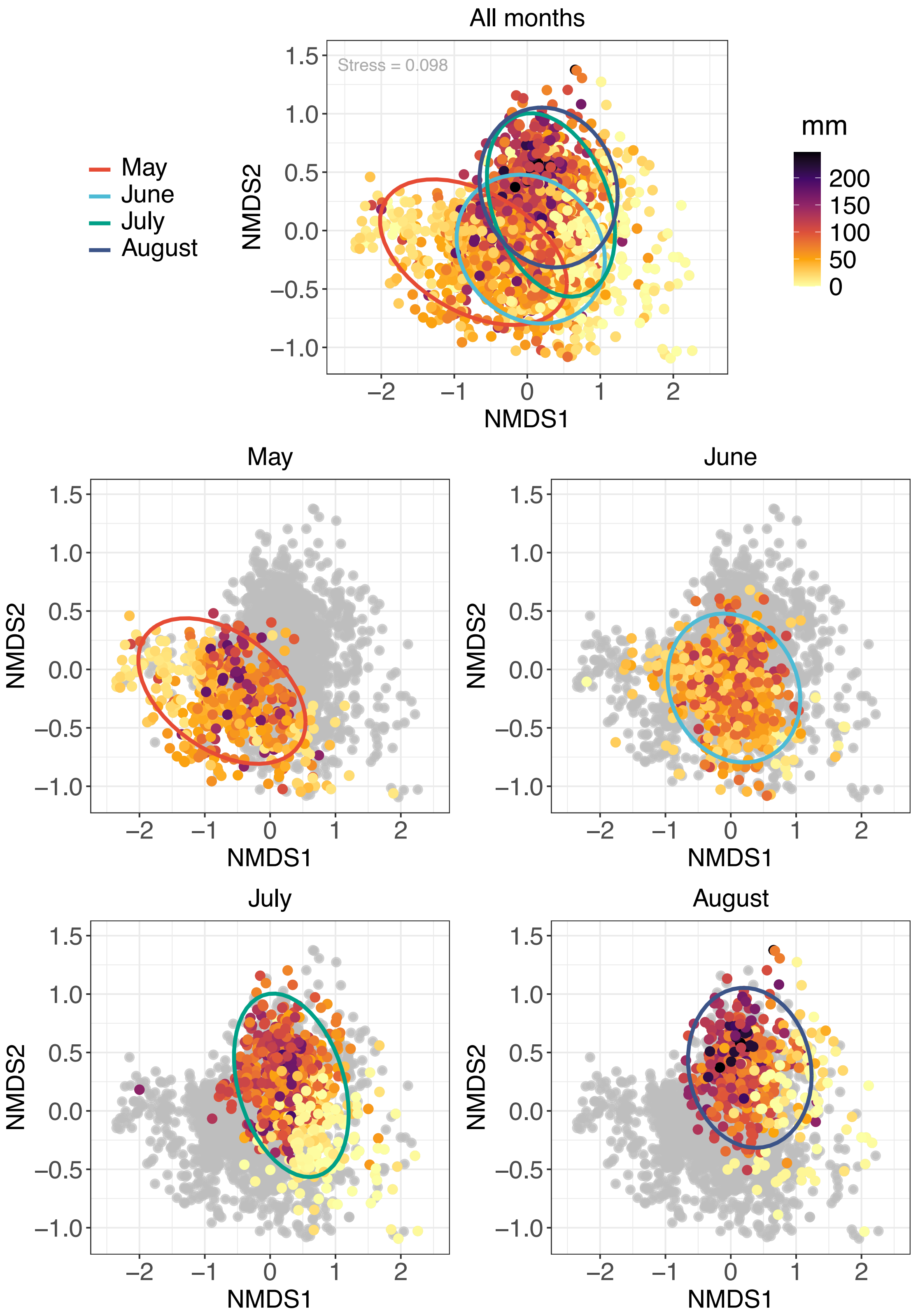

Supplementary Fig. 4. Non-metric multidimensional scaling (NMDS) of pollen composition coloured by precipitation. NMDS ordination is based on Bray-Curtis dissimilarity of n = 2 500 samples with five dimensions (k = 5); final stress = 0.098. Upper panel includes all data points, lower panels show a subset of samples from one month. NMDS was used solely for visualisation of compositional structure, and no causal interpretation was made from the ordination axes. The colour bar gradient represents the precipitation in each sample, allowing

visualisation of the difference between wetter and drier sites for the overall sampling sites and each sampling month.

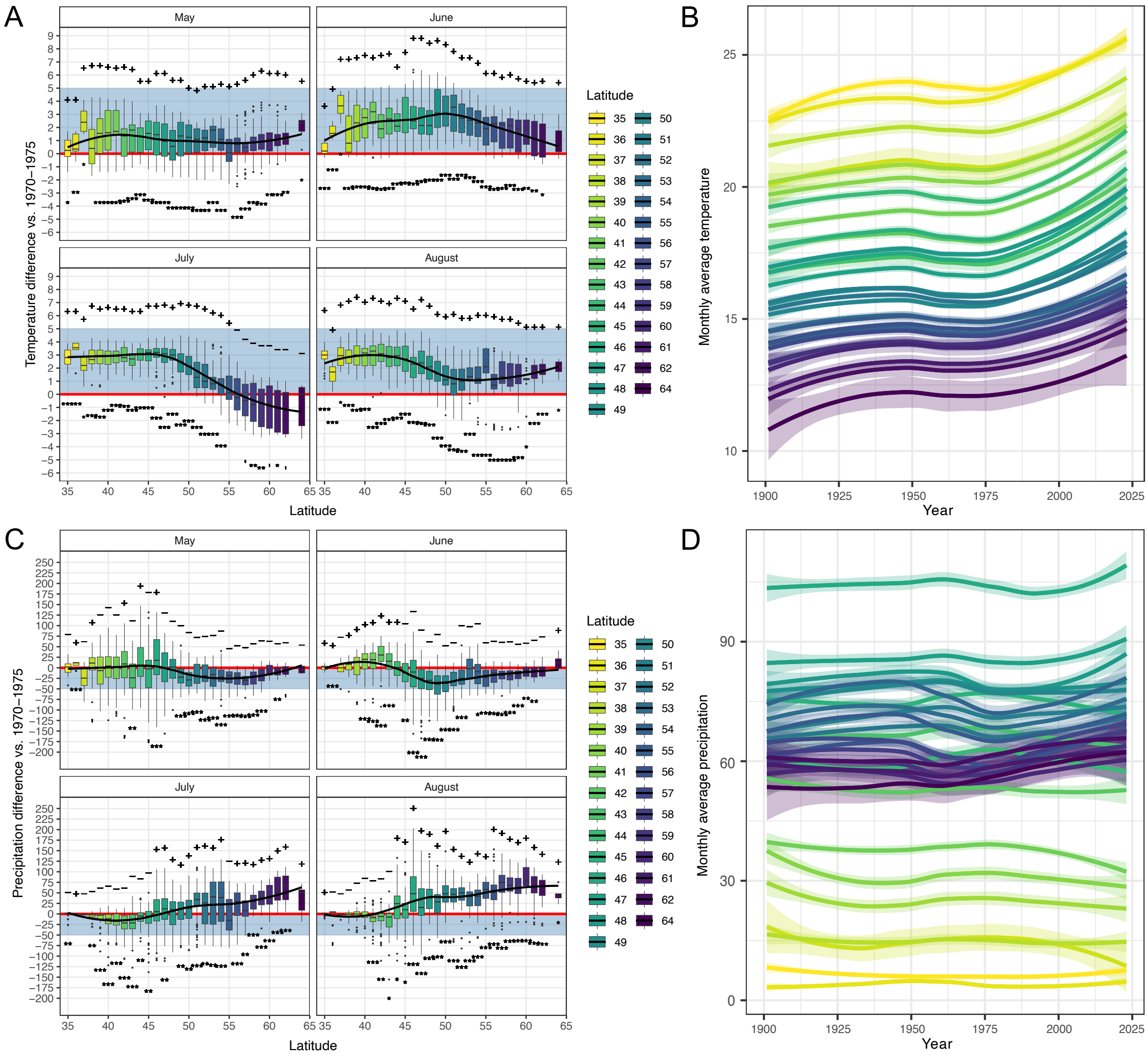

Supplementary Fig. 5. Historical development of temperature and precipitation. A and **B** illustrate temperature changes, and **C** and **D** show precipitation changes across latitudes. Blue background boxes in **A** and **C** illustrate the ranges over which climate scenarios were calculated. Error ribbons in **B** and **D** represent 95% confidence intervals. **A** Difference in mean monthly temperature between 1970-1975 versus the last 5 years (2018-2023) for each sampling site by latitude. Wilcoxon paired tests reveal significant temperature increases in Southern and Central European sites (all $p < 0.05$ listed below boxplots as *<0.05, **<0.001, ***<0.001 and direction as + = increase, and - = decrease above boxplots), with Northern latitudes showing only a trend. **B** Mean annual temperature at sampling sites from 1900 to the present, highlighting long-term trends. **C** Difference in precipitation between 1970-1975 compared to the last 5 years for each site by latitude. **D** Precipitation changes since 1900. Despite a missing trend, we observed high variation in responses of individual latitudes (range within Southern: 271.7 mm, Central: 370.5 mm, Northern: 206.8 mm), and individual latitudes always showed a strong reduction in precipitation (minimum within Southern: -124.5 mm, Central: -167.3 mm, Northern: -71.2 mm). Therefore, strong fluctuations are relevant and that our estimates of -0 to -50 mm per month are within a realistic range of occurrence, although not well predictable. For **A** and **C** boxplot boxplots are based on n = 7 074 (May: n = 1 830, June: n = 1 824, July: n = 1 770, August: n = 1 650) independent measures and visualise minimum, first quartile, median, third quartile, and maximum. The box represents the interquartile range (IQR), which contains 50% of the data points, and the whiskers extend to the smallest and largest values within 1.5 times the IQR. Outliers beyond this range are displayed as individual points. For **B** and **D** lines are based on n = 145 017 individual measures per plot and error ribbons indicate the 95% confidence interval.

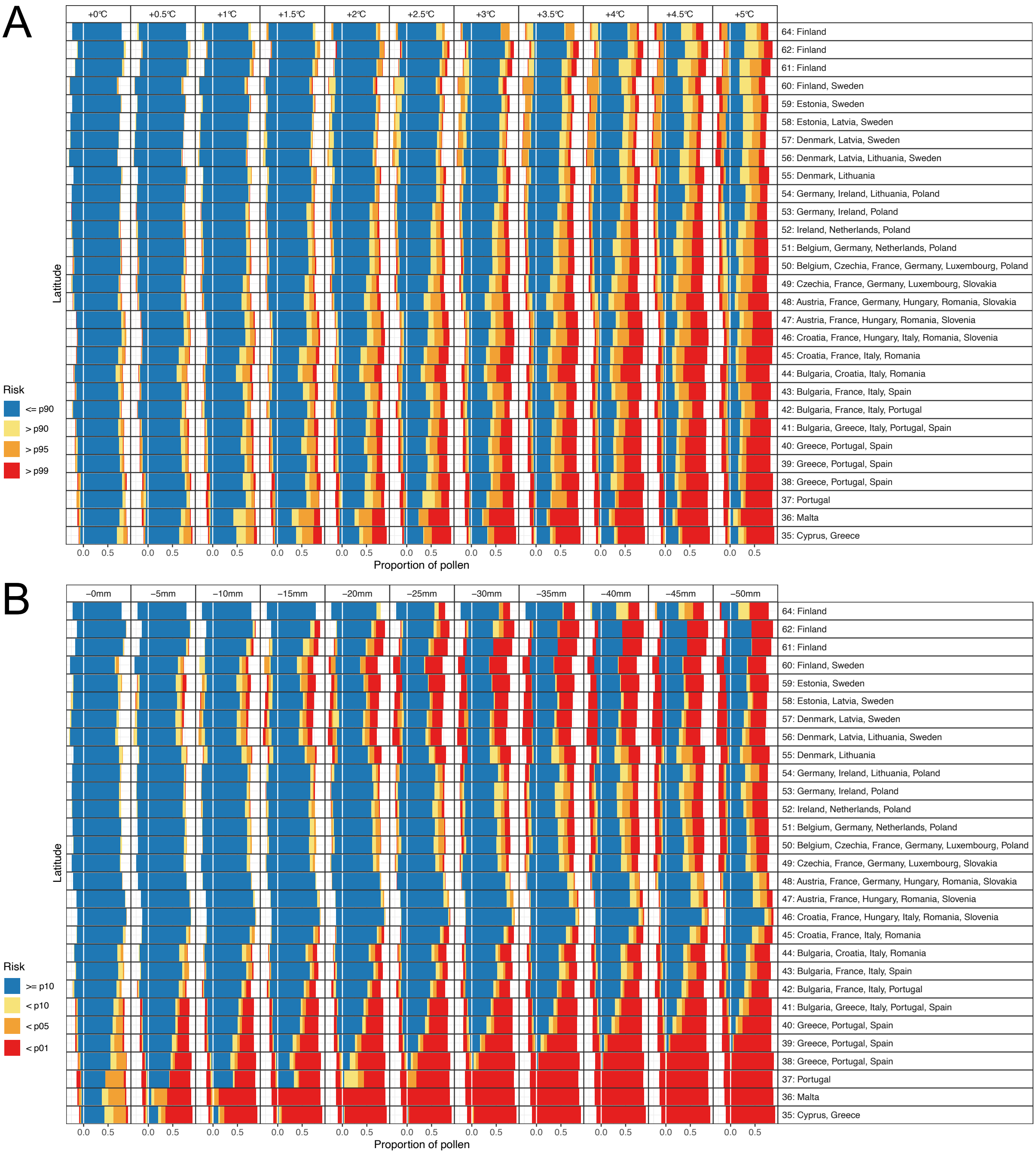

Supplementary Fig. 6. Expanded risk assessment visualisation. **A** Effect of temperature rise in 0.5 °C increments on honey bee pollen resources across different latitudes. **B** Effect of precipitation reduction by increments of 5 mm. The bar colours represent the plant species (crops and wild) that are at low risk (green), risk (yellow), high risk (orange) and critical risk (red) for several temperature scenarios (for more details, see Figure 2).

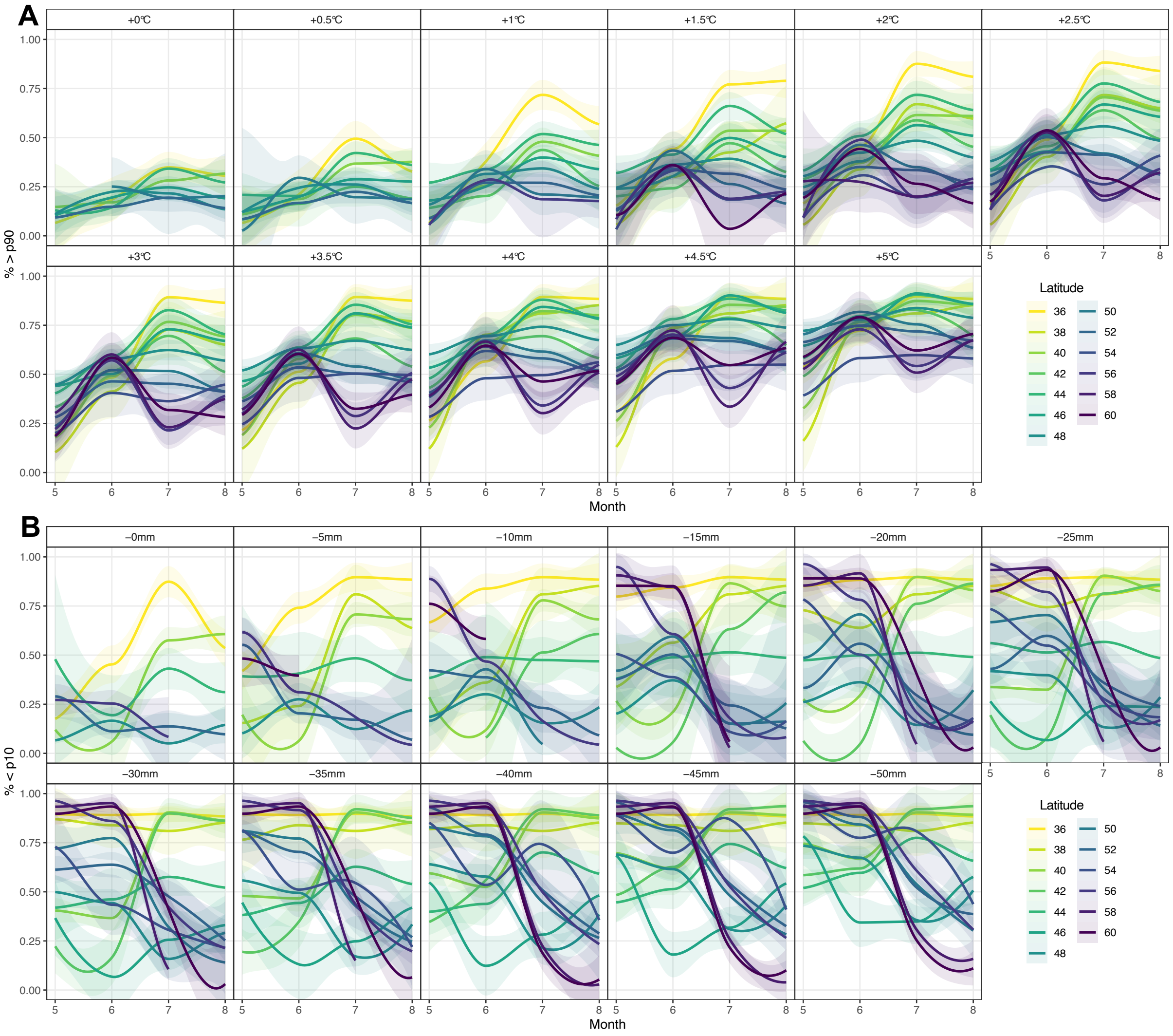

Supplementary Fig. 7. Effect of temperature rise (A) and precipitation decrease (B) on honey bee pollen resources across latitude and season. Each line represents the trend of pollen resources classified as at risk (>q90) for each latitude, and from May to August for several climate change scenarios (for more details, see Figure 3). Lighter background shades represent 95% confidence intervals. Each scenario aggregates n = 22 809 independent values.

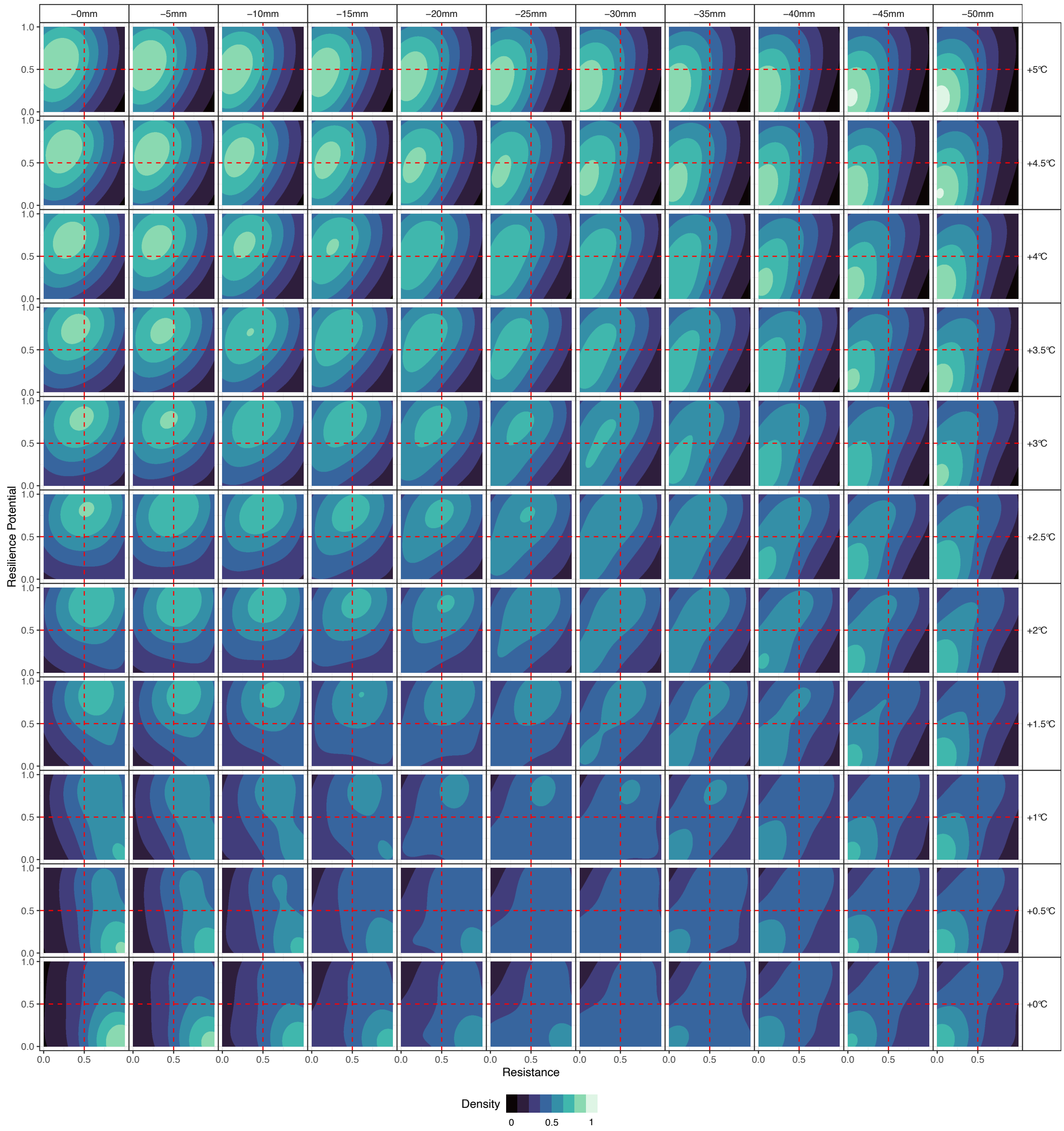

Supplementary Fig. 8. Extended plot of resistance and resilience of honey bee food resources for several climate change scenarios combining increased temperature with decreased precipitation. The plot shows the density distribution of sample resistance (the proportion of taxa not at risk at a given location and time) and sample resilience (the ability to compensate for losses in taxa at risk with other plant taxa). Each density plot aggregates *n* = 2 500 independent values. More details are provided in Figure 4.

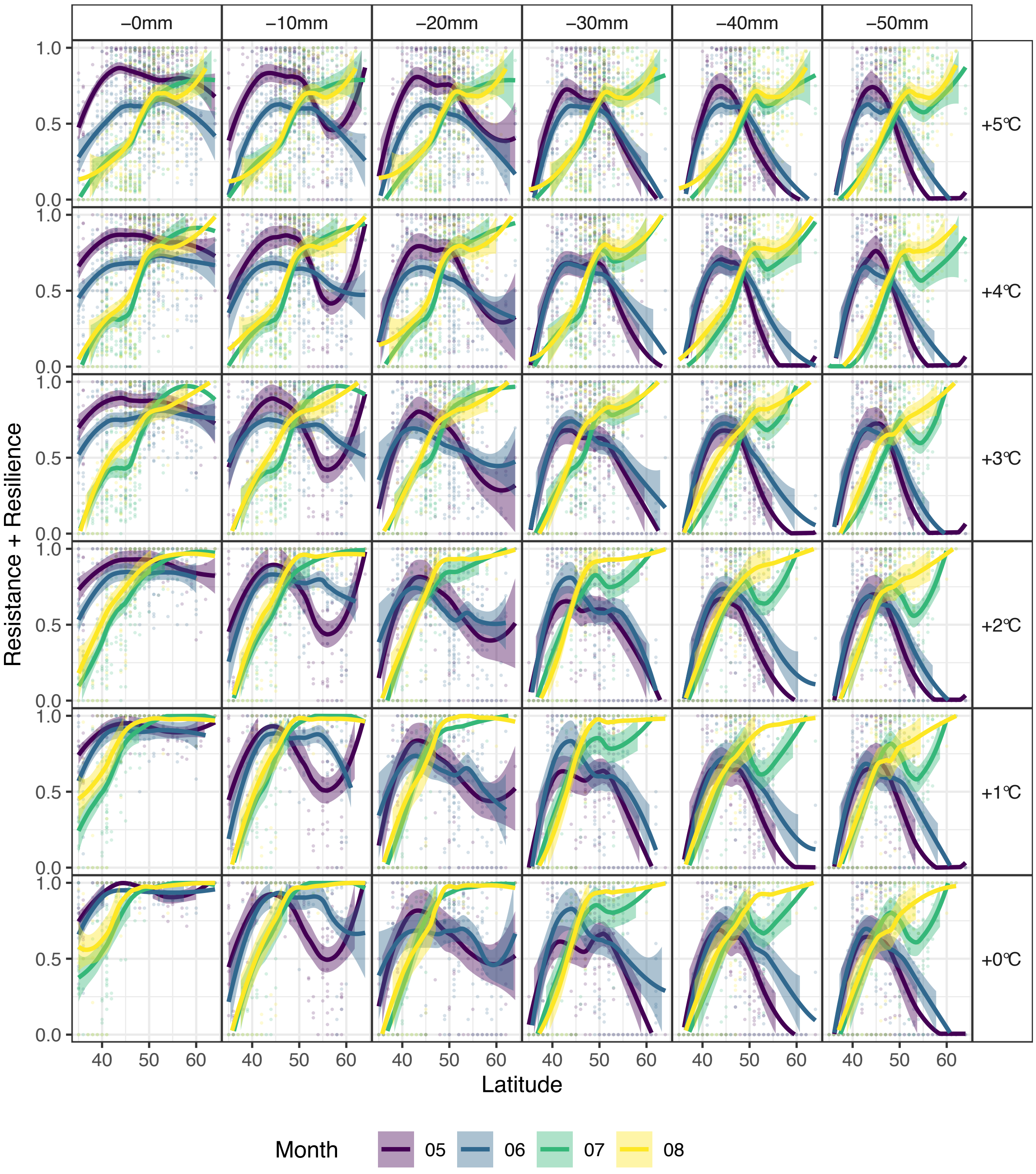

Supplementary Fig. 9. Summed values of resistance and resilience (x) across latitude (y) and season (colour) for several climate change scenarios combining decreased precipitation with increased temperature. Lighter background shades represent 95% confidence intervals. Each scenario aggregates *n* = 2 500 independent values.

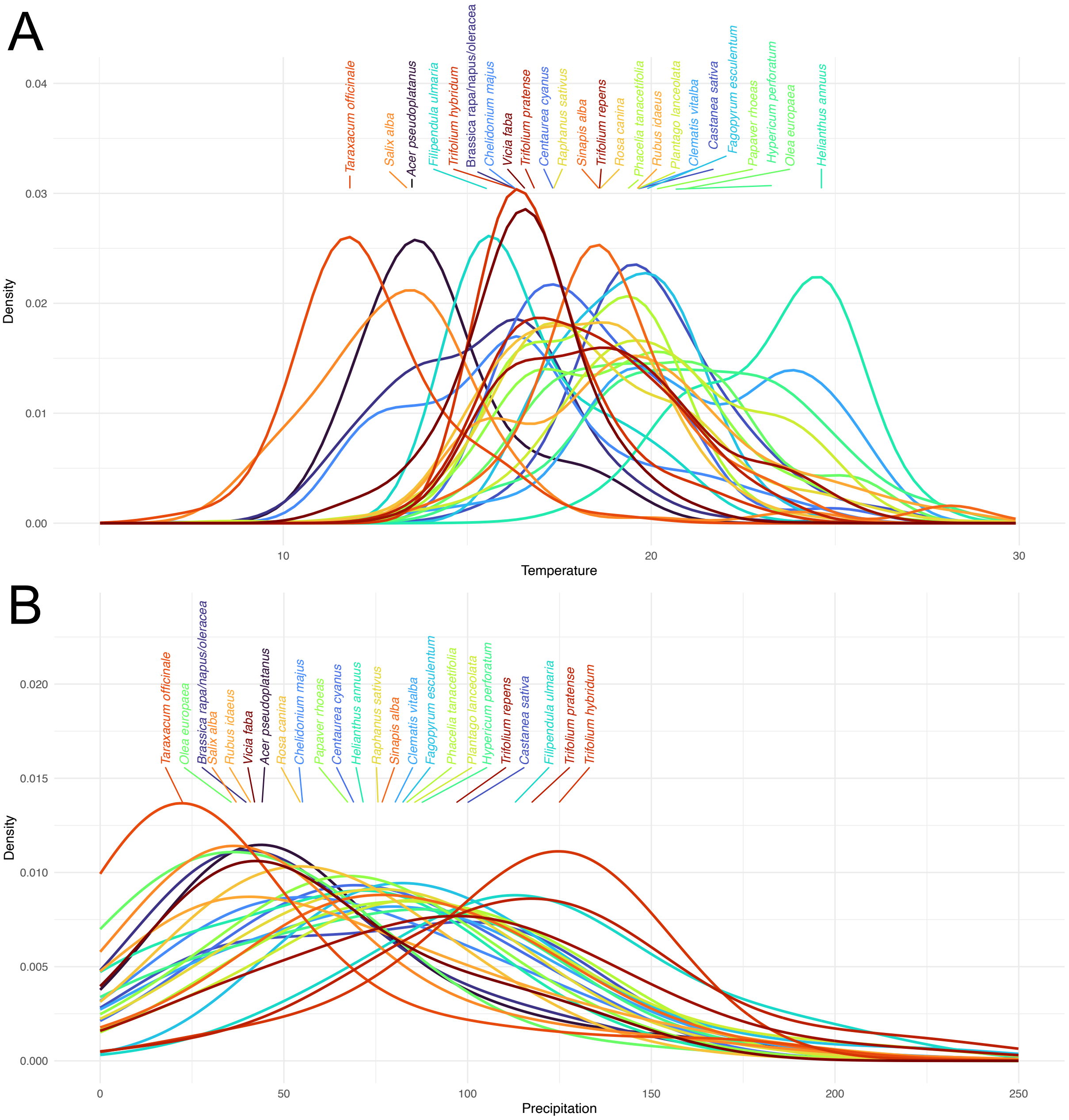

Supplementary Fig. 10. Temperature (A) and precipitation (B) response curves for the 25 most abundant plant taxa found in honey bee pollen. Curves represent abundance-weighted kernel density estimates (KDEs) constructed from empirical climate-abundance data. These KDEs provide non-parametric approximations of realised climatic associations, based on temperature and precipitation at the sites and times where each plant taxon was detected. The peak of each KDE curve reflects the most frequently encountered climatic condition for a given species.

Supplementary Table 1. Two-sided multifactorial linear model results showing the effects of climate change (temperature increase and precipitation reduction), latitude, and sampling month on honey bee food resources at risk. Latitude was tested as a quadratic term, and interaction terms are denoted by ":". Both models were significant (temperature: $F(5, 1\,189) = 100.3$, $p < 0.001$, adjusted $R^2 = 0.30$; precipitation: $F(5, 1\,138) = 56.9$, $p < 0.001$, adjusted $R^2 = 0.20$), indicating strong effects of climatic and spatial gradients on the proportion of floral resources at risk. SE=Standard Error. Denoted significance levels: ' < 0.1, * < 0.05, ** < 0.001, *** < 0.001. Exact p-values are reported unless smaller than 0.001, in which case they are given as p < 0.001.

	Temperature increase				Precipitation reduction			
	Estimate	SE	*t*	*p*	Estimate	SE	*t*	*p*
Month	1.26	0.43	2.931	0.003**	0.96	0.44	2.154	0.032*
Latitude	-0.16	0.06	-2.610	0.009**	-0.11	0.06	-1.684	0.093'
Climate change	1.95	0.58	3.373	<0.001***	3.20	0.62	5.119	<0.001***
Climate change:month	-0.03	0.06	-0.497	0.619	-0.34	0.06	-5.449	<0.001***
Climate change:latitude	-0.01	0.01	-0.831	0.406	0.00	0.00	0.053	0.958
