# Supplementary material for: Honey bee food resources under threat from climate change
Source: Nat Commun. 2025 Dec 30;17:1331. doi: 10.1038/s41467-025-68085-6 (PMC12873215; doi:10.1038/s41467-025-68085-6)
Supplement: Supplementary file 3 — Reporting Summary [file 41467_2025_68085_MOESM3_ESM.pdf]

nature portfolio

Corresponding author(s): M. Alice Pinto, Alexander Keller

Last updated by author(s): Nov 11, 2025

Reporting Summary

Nature Portfolio wishes to improve the reproducibility of the work that we publish. This form provides structure for consistency and transparency in reporting. For further information on Nature Portfolio policies, see our Editorial Policies and the Editorial Policy Checklist.

Statistics

For all statistical analyses, confirm that the following items are present in the figure legend, table legend, main text, or Methods section.

n/a	Confirmed	
☐	☒	The exact sample size (*n*) for each experimental group/condition, given as a discrete number and unit of measurement
☐	☒	A statement on whether measurements were taken from distinct samples or whether the same sample was measured repeatedly
☐	☒	The statistical test(s) used AND whether they are one- or two-sided<br>*Only common tests should be described solely by name; describe more complex techniques in the Methods section.*
☐	☒	A description of all covariates tested
☐	☒	A description of any assumptions or corrections, such as tests of normality and adjustment for multiple comparisons
☐	☒	A full description of the statistical parameters including central tendency (e.g. means) or other basic estimates (e.g. regression coefficient) AND variation (e.g. standard deviation) or associated estimates of uncertainty (e.g. confidence intervals)
☐	☒	For null hypothesis testing, the test statistic (e.g. *F*, *t*, *r*) with confidence intervals, effect sizes, degrees of freedom and *P* value noted<br>*Give P values as exact values whenever suitable.*
☒	☐	For Bayesian analysis, information on the choice of priors and Markov chain Monte Carlo settings
☒	☐	For hierarchical and complex designs, identification of the appropriate level for tests and full reporting of outcomes
☒	☐	Estimates of effect sizes (e.g. Cohen's *d*, Pearson's *r*), indicating how they were calculated

Our web collection on statistics for biologists contains articles on many of the points above.

Software and code

Policy information about availability of computer code

Data collection	The pipeline for processing raw sequencing data for metabarcoding is publicly available at GitHub chiras/metabarcoding_pipeline [https://github.com/chiras/metabarcoding_pipeline]. Code for all downstream analyses is publicly available at Zenodo 10.5281/zenodo.17578272 [https://doi.org/10.5281/zenodo.17578272] and GitHub chiras/HoneyBee-ResistanceResilience [https://github.com/chiras/HoneyBee-ResistanceResilience]. All display items presented in the main manuscript and supplementary information can be reproduced from this public data and code.
Data analysis	Data was analysed in R Studio v2024.04.2+764 using the R packages phyloseq v1.48.0, tidyr v1.3.1, speedyseq v0.5.3.9021, dplyr v1.1.4, viridis v0.6.5, bipartite v2.20, ggplot2 v3.5.1, ggsci v3.2.0, gghighlight v0.4.1, rnaturalearth v1.0.1, sf v1.0.16, geosphere v1.5.18, gdm v1.55.0.9.1, scales v1.3.0, foreach v1.5.2, doParallel v1.0.17, doSNOW v1.0.20, progress v1.2.3, patchwork v1.3.0.9000. The R code is available at https://github.com/chiras/HoneyBee-ResistanceResilience. Sequencing data was processed using the pipeline available at https://github.com/chiras/metabarcoding_pipeline. Analyses were performed on MacOSX 13.6.7 with a MacBook Pro Max M1 with 64 GB of RAM and R version 4.4.1 (2024-06-14).

For manuscripts utilizing custom algorithms or software that are central to the research but not yet described in published literature, software must be made available to editors and reviewers. We strongly encourage code deposition in a community repository (e.g. GitHub). See the Nature Portfolio guidelines for submitting code & software for further information.

Data

Policy information about availability of data

All manuscripts must include a data availability statement. This statement should provide the following information, where applicable:

- Accession codes, unique identifiers, or web links for publicly available datasets
- A description of any restrictions on data availability
- For clinical datasets or third party data, please ensure that the statement adheres to our policy

The raw sequencing data generated in this study have been deposited in the NCBI SRA database under accession code PRJNA1198597 [https://www.ncbi.nlm.nih.gov/sra/PRJNA1198597]. The processed sequencing data (for process see code availability), climate data and metadata of samples are available at Zenodo 10.5281/zenodo.17578272 [https://doi.org/10.5281/zenodo.17578272] and GitHub chiras/HoneyBee-ResistanceResilience [https://github.com/chiras/HoneyBee-ResistanceResilience]. Mean monthly temperature and monthly accumulated precipitation were extracted from the high-resolution (0.5°) gridded dataset CRU TS4.08, provided by the Centre for Climate Research at the University of East Anglia at https://catalogue.ceda.ac.uk/uuid/715abce1604a42f396f81db83aeb2a4b. All data is publicly available.

Research involving human participants, their data, or biological material

Policy information about studies with human participants or human data. See also policy information about sex, gender (identity/presentation), and sexual orientation and race, ethnicity and racism.

Reporting on sex and gender	no studies on human participants, their data or biological material were used
Reporting on race, ethnicity, or other socially relevant groupings	see above
Population characteristics	see above
Recruitment	see above
Ethics oversight	see above

Note that full information on the approval of the study protocol must also be provided in the manuscript.

Field-specific reporting

Please select the one below that is the best fit for your research. If you are not sure, read the appropriate sections before making your selection.

☐ Life sciences ☐ Behavioural & social sciences ☒ Ecological, evolutionary & environmental sciences

For a reference copy of the document with all sections, see nature.com/documents/nr-reporting-summary-flat.pdf

Ecological, evolutionary & environmental sciences study design

All studies must disclose on these points even when the disclosure is negative.

Study description	We analysed 2,500 pollen samples collected by honeybees biweekly between May and August 2023 from 310 apiaries distributed across all 27 EU countries. We identified the pollen composition of these samples using DNA metabarcoding of the internal transcribed spacer 2. DNA metabarcoding was carried out in triplicates that were then combined for data precess.
Research sample	We chose Apis mellifera, the honeybee, as a model, due to its ubiquity, importance as a pollinator in agroecosystems and generalised foraging, to reflect the impacts of climate change on bee resource diversity.
Sampling strategy	315 apiaries were chosen based on apicultural activities, with the number of apiaries ranging from 5 to 20 per country proportional to the surface area. 5 beekeepers gave up at the beginning of the sampling though. No formal sample size calculation was performed, as the dataset represents an unprecedented and balanced EU-wide coverage sufficient for robust statistical analyses.
Data collection	Pollen was collected from traps placed, for 1 day, in front of 2 beehives entrance. The beekeepers collected 5g in total of pollen from both hives to the same recipient stored with silica bags to dry and preserve the pollen. A ID code containing the sampling number, the sampling site was already provided. Beekeepers only needed to add the date of collection using a pencil.
Timing and spatial scale	The sampling went from the first week of May until the last week of August of 2023. The pollen was collected every 15 days for a total of 9 samplings, across the 27 European Union countries.
Data exclusions	No data was excluded
Reproducibility	Positive controls were added to each batch of sampling to assure the reproducibility. Negative controls were also added to assure the

Reproducibility	lack of contamination during the laboratorial process. Statistical analyses were rerun on Microsoft windows and Ubuntu, with different R versions to ensure interoperable reproducibility.
Randomization	Apiaries were chosen based on apicultural activities, willingness of citizen scientists and the surface area of a country.
Blinding	Blinding was not possible in our study. Blinding methods are not established for field studies on biodiversity and not relevant for sampling data of pollen composition at different locations, since there are no subjective judgements involved that may bias the results across sites.

Did the study involve field work? ☒ Yes ☐ No

Field work, collection and transport

Field conditions	Pollen collection activity by honey bees is influenced by environmental conditions, particularly temperature and precipitation. Sampling was therefore conducted during favourable weather, as low temperatures and rainfall reduce bee foraging activity and pollen availability.
Location	The study was done at a European Union scale. Ranging from latitude 35º to 64º, and from longitude -9º to 34º, and comprising the countries Austria, Belgium, Bulgaria, Croatia, Cyprus, Czechia, Denmark, Estonia, Finland, France, Germany, Greece, Hungary, Ireland, Italy, Latvia, Lithuania, Luxembourg, Malta, Netherlands, Poland, Portugal, Romania, Slovakia, Slovenia, Spain, and Sweden. More information can be found in the metadata file available at https://github.com/chiras/HoneyBee-ResistanceResilience
Access & import/export	Beekeepers collected the samples following a manual developed by the INSIGNIA consortium in compliance with local, national and international laws. The samples would be sent to each country National Coordinator, which in turn would sent the package to our laboratory. No permit was necessary since pollen was transported in vials with silica bags only within the EU.
Disturbance	no disturbance was caused by the study

Reporting for specific materials, systems and methods

We require information from authors about some types of materials, experimental systems and methods used in many studies. Here, indicate whether each material, system or method listed is relevant to your study. If you are not sure if a list item applies to your research, read the appropriate section before selecting a response.

Materials & experimental systems

n/a	Involved in the study	
☒	☐	Antibodies
☒	☐	Eukaryotic cell lines
☒	☐	Palaeontology and archaeology
☒	☐	Animals and other organisms
☒	☐	Clinical data
☒	☐	Dual use research of concern
☐	☒	Plants

Methods

n/a	Involved in the study	
☒	☐	ChIP-seq
☒	☐	Flow cytometry
☒	☐	MRI-based neuroimaging

Dual use research of concern

Policy information about dual use research of concern

Hazards

Could the accidental, deliberate or reckless misuse of agents or technologies generated in the work, or the application of information presented in the manuscript, pose a threat to:

No	Yes	
☒	☐	Public health
☒	☐	National security
☒	☐	Crops and/or livestock
☒	☐	Ecosystems
☒	☐	Any other significant area

Experiments of concern

Does the work involve any of these experiments of concern:

No	Yes	
☒	☐	Demonstrate how to render a vaccine ineffective
☒	☐	Confer resistance to therapeutically useful antibiotics or antiviral agents
☒	☐	Enhance the virulence of a pathogen or render a nonpathogen virulent
☒	☐	Increase transmissibility of a pathogen
☒	☐	Alter the host range of a pathogen
☒	☐	Enable evasion of diagnostic/detection modalities
☒	☐	Enable the weaponization of a biological agent or toxin
☒	☐	Any other potentially harmful combination of experiments and agents

Plants

Seed stocks	no seed stocks were used in the study
Novel plant genotypes	no novel plant genotypes were used in the study
Authentication	no authentication was required
